# Investigation of Red Blood Cells by Atomic Force Microscopy

**DOI:** 10.3390/s22052055

**Published:** 2022-03-07

**Authors:** Viktoria Sergunova, Stanislav Leesment, Aleksandr Kozlov, Vladimir Inozemtsev, Polina Platitsina, Snezhanna Lyapunova, Alexander Onufrievich, Vyacheslav Polyakov, Ekaterina Sherstyukova

**Affiliations:** 1 Laboratory of Biophysics of Cell Membranes under Critical State, Federal Research and Clinical Center of Intensive Care Medicine and Rehabilitology, V.A. Negovsky Research Institute of General Reanimatology, 107031 Moscow, Russia; va.inozemcev@physics.msu.ru (V.I.); snezhanna.lyapunova@yandex.ru (S.L.); kmanchenko@yandex.ru (E.S.); 2 NT-MDT Spectrum Instruments, Proezd 4922, 4/3 Zelenograd, 124460 Moscow, Russia; leesment@ntmdt-si.com (S.L.); polyakov@ntmdt-si.com (V.P.); 3 Department of Medical and Biological Physics, Sechenov First Moscow State Medical University (Sechenov University), 119991 Moscow, Russia; fillnoise@mail.ru; 4 Institute of Biotechnical Systems and Technologies National Research“MIET”, Shokin Sq., Build.1, 124498 Zelenograd, Russia; platitsina@ntmdt-si.com; 5 Federal State Budgetary Institution “N.N. Burdenko Main Military Clinical Hospital” of the Ministry of Defense of the Russian Federation, Hospital Sq., Build. 3, 105094 Moscow, Russia; alonuf@gmail.com

**Keywords:** imaging, atomic force microscopy, red blood cells, morphology, nanostructure, cytoskeleton, membrane stiffness

## Abstract

Currently, much research is devoted to the study of biological objects using atomic force microscopy (AFM). This method’s resolution is superior to the other non-scanning techniques. Our study aims to further emphasize some of the advantages of using AFM as a clinical screening tool. The study focused on red blood cells exposed to various physical and chemical factors, namely hemin, zinc ions, and long-term storage. AFM was used to investigate the morphological, nanostructural, cytoskeletal, and mechanical properties of red blood cells (RBCs). Based on experimental data, a set of important biomarkers determining the status of blood cells have been identified.

## 1. Introduction

Atomic force microscopy (AFM) is a cutting-edge technology that can be used to study the morphology and biomechanical properties of blood components. The advantages of AFM over other microscopy techniques include high resolution, the possibility of using various media, including a native solution for living cells, and direct measurement of cell mechanical properties. The AFM operates by “touching” the surface of the sample using a tip probe with control of interaction force [1,2,3,4,5].

This method does not require additional sample modification such as freezing, metal plating, or the use of vacuum or dyes. AFM works both in air and in a liquid environment, so samples can be studied in their physiological buffer solutions.

Molecular-level resolution of soft samples can be achieved during scanning [4,5]. It reveals the morphology of individual biomolecules that cannot be seen using other imaging methods. Depending on the purpose of the study, semi-contact, contact, or jumping modes of investigation can be used [6,7].

The essential element of AFM is a probe sensor which represents a cantilever with a sharp probe at the end. The probe tip diameter may vary from 1 to 10,000 nm depending on the application. The probe is usually made of crystalline silicon or silicon compounds [8].

Visualization of cells using AFM is limited by the very soft and volatile nature of the cell surfaces. To visualize red blood cells (RBCs) in liquid, it is necessary to prepare a sticky substrate, such as one with polylysine, and fix the RBCs [6,9]. Notably, fixation of cell samples can cause several artifacts resulting in membrane surface alterations [10,11,12]. If the samples are poorly attached to the surface, they will be displaced by the AFM tip during imaging.

The dimensions of the object vary greatly depending on the preparation of the sample. In liquid, the height of the control discocyte is about 4.5 times the height of the air-dried erythrocyte. Topographic AFM imaging can provide direct, high-resolution morphological observation of cells. AFM, with its high-resolution imaging, allows quantitative analysis of imaging parameters such as nanostructure profile, pore size, and clustering of RBC cytoskeleton filaments [13]. Nanostructure and defect size are parameters that have been used to quantify the complexity of cell membrane structure.

Atomic force spectroscopy (AFS) allows us to study the mechanical properties of cells based on experimental force curves *F*(*h*), i.e., the relationship between the cantilever vertical bending and the vertical motion of the piezoelectric scanner is assessed [13,14,15,16,17,18]. AFM and AFS both represent direct real-time observation of biological objects.

RBCs can be an important target for pathogenic factors. These cells can serve as a link between the impact of a pathogenic factor on a biological system and its clinical response [19].

The cytoskeleton, a structural molecular component of RBCs that controls their morphology, membrane deformability, and nanostructure [20,21], determines their functional status as well as particularly their capacity to deliver oxygen to the tissues. The study of the RBC cytoskeleton has important scientific and clinical implications.

Electron microscopy made it possible for the first time to reveal the configuration of the RBC cytoskeleton [22,23,24]. However, sample preparation for this method impairs the integrity of objects and changes their structure. In actual studies, atomic force microscopy is used to visualize blood cells and study their membranes [25,26,27,28].

AFM allows quantitative measurements of the membrane surface, as well as the study of morphological structural characteristics and mechanical properties under various diseases [1,29,30,31,32,33,34], and the impact of exposure to physical and chemical factors [35,36,37,38].

AFM and AFS expand significantly the amount of information about RBCs since both methods are suitable for studying the characteristics of living cells in buffer at the nano level.

In this study, we would like to reveal the prospects of using the atomic force microscope, a promising diagnostic technique for blood cells.

## 2. Materials and Methods

### 2.1. Atomic Force Microscopy

**Figure 1:**
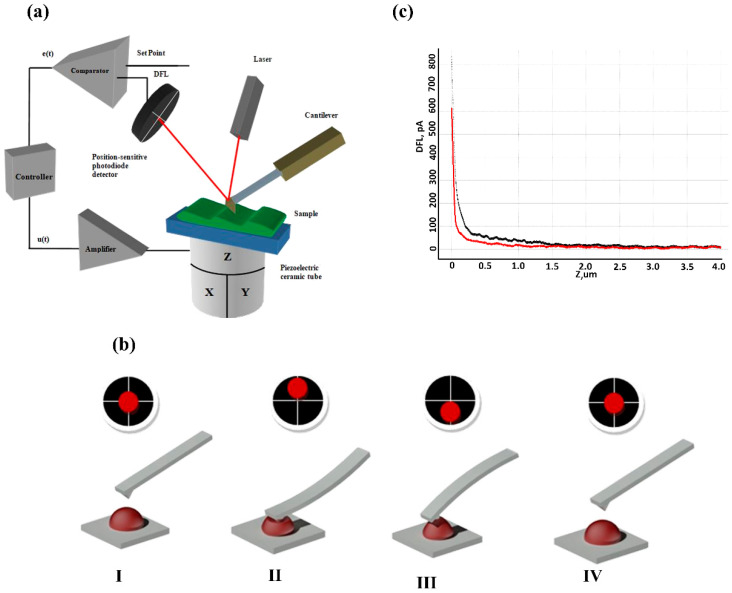
The principles of the atomic force microscopy operation. (**a**) Block diagram of AFM; (**b**) Measurement of blood cell membrane stiffness by AFM. I: the scanner is brought to the cell membrane; the laser spot is located in the center of the photodiode. II: the probe indents the cell membrane; the laser spot moves up relative to the center (located in the upper sections). III: the probe is detached from the cell membrane; the laser spot moves down relative to the center (located in lower sections). IV: the piezo scanner is taken away from the cell; the laser spot is located in the center of the photodiode. (**c**) Empirical force curve. The forward motion is black, the reverse motion is red.

The atomic force microscope, NTEGRA Prima (NT-MDT Spectrum Instruments, Moscow, Zelenograd, Russia) was used to study the cells and their cytoskeleton. The atomic force microscope operational principle is based on the measurement of Van der Waals interaction forces between the sample surface. A block diagram of AFM is shown in Figure 1a. The object under study was the surface of a sample fixed on a precision triaxial piezoelectric transducer (scanner). The sensor is a sharp silicon probe fixed on a flexible cantilever. The cantilever vertical deflection is proportional to the interaction force between the needle and the sample. A laser beam is focused on the cantilever surface and is reflected on a sectioned photodiode. Thus, the displacement of the reflected beam on the photodiode is also proportional to the interaction force between the probe and the sample. The signal from the photodiode enters the comparator, which compares it with the desired interaction force set by the operator. The comparator outputs an error signal that reaches the feedback control unit. The control signal on the feedback control output goes to the high-voltage amplifier input, thus controlling the length of the Z component of the piezoelectric scanner. By registering the Z coordinate and other signals related to the interaction forces between the probe and the sample during the precise movement of the sample in the XY plane, we can record the surface topology and other characteristics with a high spatial resolution.

Imaging of the surface morphology was performed in air using a semi-contact mode by silicon cantilevers (NSG01 series) with a gold reflective coating (NT-MDT Spectrum Instruments, Moscow, Zelenograd, Russia).

The height of the cantilever tip was 14–16 µm, and the curvature radius of its apex was 10 nm. The typical resonant frequency was 148 kHz, the force constant *K* was 5 N/m.

Depending on the task, the scanning fields were chosen from a maximum of 100 × 100 μm^2^ and reduced down to 0.25 × 0.25 μm^2^. The number of points per scan was 512 or 1024, the scanning rate varied from 0.3 to 0.9 lines per second. The obtained images and their profiles were analyzed as 2D and 3D AFM images.

### 2.2. Atomic Force Spectroscopy

AFM, in addition to obtaining nanoscale images, can measure biomechanical characteristics of biological structures by atomic force spectroscopy. For this purpose, the probe is placed over the studied object. Mechanical properties are measured in liquid (PBS, pH 7.4). The operator sets the desired interaction force, which causes the probe to indent (Figure 1b) the surface of an object, for example, a membrane. The deflection response shape is determined by the combination of biomechanical properties of an object, in particular, its Young’s modulus, the specified force, and the probe size. The Hertz model was used to calculate Young’s modulus.

To measure the mechanical properties, it is important to consider the cantilever spring constant K_c_ and the stiffness of the cell membranes at a load of interest. The spring constant of the selected cantilever K_c_ should be chosen based on two considerations: K_c_ should be much higher than the object stiffness (K_m_) to ensure the dominant role of indentation during the force spectroscopy. At the same time, K_c_ should be small enough to provide sufficient sensitivity at h_Hz_ penetration range. K_c_ around 1 N/m met both these criteria.

So, the optimal value of K for dry cell membranes was about 10–50 N/m, and for live cells, it was 0.05–1.8 N/m. Based on this, we used cantilevers SD-R150-T3L450B-10 (Nanosensors, Neuchâtel, Switzerland) with K = 1 N/m and R = 150 nm. For the calibration of the cantilever spring constant, the thermal tune method was applied.

The empirical force curve (Figure 1c) represents the relationship between the deflection current of the photodiode I and the vertical displacement Z of the piezo scanner. These parameters can be varied during AFM measurements depending on the purpose and object of the study). For our experiment, the maximum value of piezo scanner displacement Z_max_ was 4000 nm and the force value was 60 nN. The study was conducted at a vertical speed of less than 1 μm/s. Stiffness calculations were performed at a until a depth of h_Hz_ [39]. h_Hz_ is the depth of homogeneous indentation and probe immersion, until which the F(h) function can be described using the Hertz model. More detailed information on the measurement process and probes is described earlier in [39].

### 2.3. Monolayer of Cells for Studying Morphology and the Nanosurface of Red Blood Cell Membranes

Experiments were performed using packed red blood cells (pRBCs) in sealed containers with CPD (citrate-phosphate-dextrose) anticoagulant and SAGM (saline-adenine-glucose-mannitol) resuspension media. The pRBCs were obtained from blood transfusion centers in Moscow, Russia, and stored at 4 °C. Cells stored for 2–6 days served as a control.

A V-Sampler (Vision, Vienna, Austria) was used to obtain a monolayer of cells. Slides were placed on holders and 10 µL of the sample was applied. The samples were spread on the slide using glass covers.

The cell membrane surface is a complex heterogeneous structure. We used the FemtoScan Online (Femtoscan, Moscow, Russia) software to detect small structural changes, quantify them at different scales and perform a statistical comparison of their sizes during storage of pRBCs and on exposure to pathogenic factors. Using the spatial Fourier transform, we decomposed a complex AFM image of the nanosurface into two simpler ones with different spatial resolutions. The spectral window with low spatial frequencies contains images of membrane structures with a large spatial period (L_1_, h_1_), while the spectral window with high frequencies provides images of membrane structures with a small spatial period (L_2_, h_2_). Parameters of spatial scales were chosen according to the natural structures of RBC membranes. Nanosurface decomposition was performed in several stages. A more detailed description of the spatial Fourier transform technique is given in our previous studies [13,40].

### 2.4. Red Blood Cells Cytoskeleton

To obtain images of the cytoskeleton network and its individual constituents, RBC ghosts were created. Sample preparation was carried out in several stages. At the first stage, the removal of components of the solution where the red blood cells were stored was done. For this, 500 μL of phosphate-buffered saline with pH 7.4 was added to 100 μL of RBC suspension. Then the suspension was stirred in Mini-Rotator Bio RS-24 (SIA BIOSAN, Riga, Latvia)at 8 rpm for 5 min. Cell sedimentation was done at the second stage. For this purpose, the suspension was centrifuged at 1900 rpm for 5 min in a Universal 320 centrifuge(Andreas Hettich GmbH & Co., KG, Tuttlingen, Germany). The supernatant was removed and 500 µL of buffer was added. The stirring and settling process was repeated 5 times. After that, 75 µL of precipitated cells were left. The next step was hemolysis of the obtained RBCs. For this purpose, 500 μL of 0.09% NaCl in distilled water was added to the cells. The resulting suspension was centrifuged at 1900 rpm for 5 min. The supernatant was then removed. Then, 300 μL of distilled water was added to the suspension for further hemolysis, the suspension was stirred for 5 min in a Bio RS-24 rotator (8 rpm, 5 min) and left in the refrigerator at 4 °C for 30 min and then at 24 °C for 10 min for further hemolysis. RBC ghost formation was observed in the resulting sample. A monolayer was prepared from the precipitate using a V-Sampler device for AFM scanning.

### 2.5. Sample for Obtaining Force Curves Using Atomic Force Spectroscopy

It was necessary to study the mechanical properties of erythrocyte membranes in a liquid medium because only in this case living object characteristics could be obtained. However, this condition imposes the limitation for high-resolution imaging.

In our nanomechanical studies of native RBCs, the sample was prepared according to the following procedure. First, 100 μL of pRBCs was added to 500 μL of phosphate-saline buffer with pH 7.4. Then, by triple centrifugation at 1500 rpm for 5 min, the rest of the preservative solution was removed. To prepare the suspension, 50 µL of erythrocytes was added to 10 mL of phosphate buffer. 200 μL of the suspension was applied to a coverslip coated with polylysine solution [41]. Erythrocytes were left for 30 min to adhere to the glass. The obtained sample was washed in phosphate-saline buffer with pH 7.4 for 10 s and then measurements were performed in a liquid cell using AFM.

### 2.6. Membrane Pathogenic Factors

Hemin, Zn^2+^, and red blood cells suspension of long-term storage were used as membrane pathogenic factors.

In the first series of experiments, dry hemin (Sigma-Aldrich, Saint Louis, MO, USA)was used. 100 mg of NaOH was dissolved in 5 mL of distilled water. Next, 25 mg of dry hemin was dissolved in 0.5 mL of the solution prepared earlier, and 2.5 mL of distilled water was added. The resulting solution was added in a volume of 50 μL to the tubes with pRBC suspension. The exposure time was 15 min.

In the second series of experiments, Zn^2+^ was used. 90 mg of ZnSO_4_ (Sigma-Aldrich, Saint Louis, MO, USA)was dissolved in 5 mL of phosphate buffer with pH 7.4. Then, 10 μL of the resulting solution was added to 100 μL of pRBC. The concentration of Zn^2+^ ions was 4 mM.

For the pRBC storage experiments, a sample was taken from a 13 mL pRBC bag on days 2–6 and 31.

### 2.7. Statistical Analysis

Cell monolayer

After exposure of blood cell membranes by modifiers, 3 areas of 100 × 100 μm^2^, 50 × 50 μm^2^, and 20 × 20 μm^2^ were scanned using AFM for each sample. For erythrocyte nanostructure analysis, 2.5 × 2.5 μm^2^ areas on 5 cells were scanned for each sample. 280 red blood cells images were scanned in total.

Ghost monolayer

A 2.5 × 2.5 μm^2^ area was scanned in 30 RBC ghosts to analyze the nanostructure of the cytoskeleton. With a pixel resolution of 1024 × 1024 pixels, 180 areas in the ghosts were analyzed in total.

Measurement of Young’s modulus

About 100 native cells were measured at each sample in the experiments. One force curve per cell was constructed. About 400 native RBCs were analyzed.

The statistical analysis of the results was performed using OriginPro 2019 software (OriginLab Corporation, Northampton, MA, USA). Statistical analysis included calculation of the mean, standard deviation, histograms, and distribution functions. Histogram approximation based on normal data distribution was performed. Nonlinear approximation of empirical force curves was also performed using the Origin Pro 2019 software. A one-way ANOVA test followed by the post hoc Tukey’s test for experimental data comparison was used.

## 3. Results

### 3.1. Morphology and Nanostructure of RBC Exposed to the Chemical and Physical Factors

**Figure 2:**
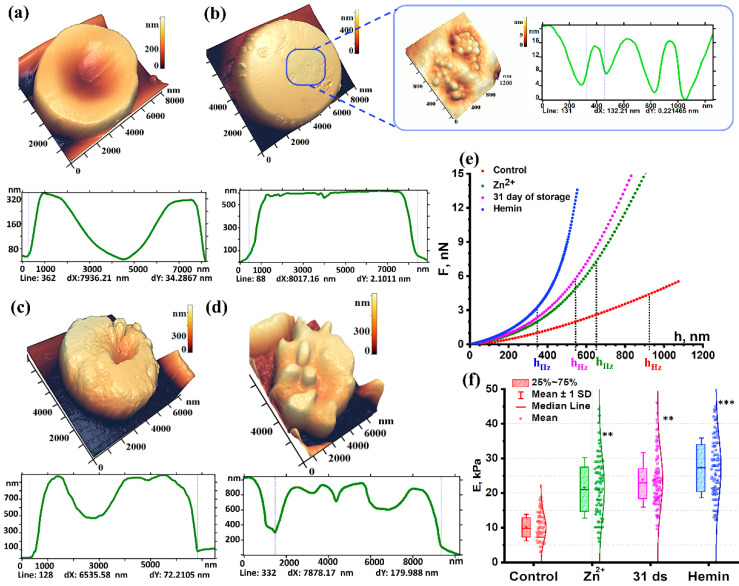
AFM image of the red blood cell (RBC) morphology (10 × 10 µm^2^) in air and their profiles, respectively: (**a**) Control; (**b**) RBC after exposure to hemin, 3D AFM image of a 2000 × 2400 nm^2^ area with granular structures after exposure to hemin and domain structure profile; (**c**) RBC after exposure to zinc ions; (**d**) RBC on day 31 of storage; (**e**) Force curves of RBCs in liquid on exposure to different agents, the curves are the average over all the curves for a specific condition; (**f**) Change in Young’s modulus depending on pathogenic factors. Data are presented as box plots. One-way ANOVA test followed by post hoc. Tukey was used: ** *p* < 0.01, *** *p* < 0.001 compared to control.

Morphology is one of the important indicators of RBC status. Cell morphology was studied after exposure to pathogenic factors. AFM images of RBCs exposed to these factors are shown in Figure 2.

Normally, the RBC diameter is 7900 ± 800 nm. The maximum height of the discocyte torus is 320 ± 80 nm (Figure 2a). The profile marker (dotted line on the cell surface) is drawn to show the RBC concavity and estimate its height. Packed RBCs stored for 2–6 days were taken as control. When the profile was evaluated using the spatial Fourier transform method, L_1_ of the membrane nanosurface was 1133 ± 150 nm, L_2_ was 90 ± 10, and the roughness height h_1_ did not exceed 5.6 ± 1.5 nm, while h_2_ was 1.1 ± 0.5 nm. The data are presented in Table 1. It shows the L_1_, h_1_, L_2_, h_2_ values as mean ± standard deviation, the ratios of the mean and the control values are given in parentheses.

Hemin is a natural oxidizing agent of biological structures that disrupts the conformation of spectrin, the band 4.1 protein, and loosens the bond between them [35]. The formation of leptocytes with an altered surface is one of the common effects of hemin (Figure 2b). Topological defects appeared on the membrane as domains with an internal grain-like structure. The size of one domain was 500 ± 210 nm. The individual grain size was 130 ± 40 nm. Thus, hemin disrupted the cytoskeleton, which manifested as altered intrinsic parameters of the h_2_ nanosurface.

Zinc ions are known to be a vital trace element that participates in cell growth and division as well as in protein and nucleic acid synthesis [42]. An excess of zinc ions can contribute to RBC lysis and anemia. This ion induces the cluster of band 3 proteins [41].

Upon exposure of RBCs to ZnSO4, the cells transformed into stomatocytes with a crumbly surface (Figure 2c). Its profile size is about 6500 nm.

On day 31 of pRBCs storage, 71 ± 7% of echinocytes and spheroechinocytes were observed in the monolayer. A typical echinocyte on day 31 of storage is shown in Figure 2d. Its profile size is about 7900 nm. The following dimensions of the spicules on the cell surface were observed: width L up to 1500 nm and height h up to 350 nm.

As is evident from the data presented, the chemical factors such as the presence of hemin and zinc had the strongest effect on h_1_, L_2,_ and h_2_.

The h_1_ values differed significantly across all studied groups. Thus, hemin increased its value by 3.3 times, zinc by 9.8 times, and storage by 1.6 times (Table 1).

### 3.2. Young’s Modulus after Exposure to Pathogenic Factors

Young’s modulus was calculated at h_Hz_ depth for all experimental curves. h_Hz_ depth represents the threshold of the Hertz model applicability [39]. When its value is reached, the deformability of RBCs under external impact with preserved integrity can be assessed.

Analysis of the force curves showed the value of Young’s modulus of the control RBC membranes at a level of 10 ± 4 kPa (Figure 2f). Upon exposure to hemin, Young’s modulus E increased to 28.1 ± 7.5 kPa in comparison to the control.

Upon exposure to zinc, Young’s modulus for RBC membranes in 15 ± 2% of the total cells remained at the control level. The stiffness of the rest of the RBCs increased 2.4 and more times. The Young’s modulus E_average_ was 23.7 ± 9.7 kPa (*p* < 0.01) vs the control.

Evaluation of the mechanical properties of erythrocyte membranes during storage of pRBCs has shown that Young’s modulus increased by day 31 of storage up to 24 ± 9 kPa (*p* < 0.01). The proportion of cells with Young’s modulus at the control level was 18 ± 4%. Thus, the stiffness of RBC membranes during storage of pRBCs increased by 2.4 ± 0.2 times (Figure 2f).

On exposure to the modifying factors, the h_Hz_ parameter values changed. Its value in the control group was 920 ± 310 nm, for the Zn^2+^ exposure group it reached 625 ± 230 nm, while after 31 days’ storage it dropped to 570 ± 180 nm, and for hemin exposure even further to 380 ±190 nm (Figure 2e).

### 3.3. Structural Parameters of Cytoskeleton during Long-Term Storage of pRBC and Exposure of RBCs to Hemin

**Figure 3:**
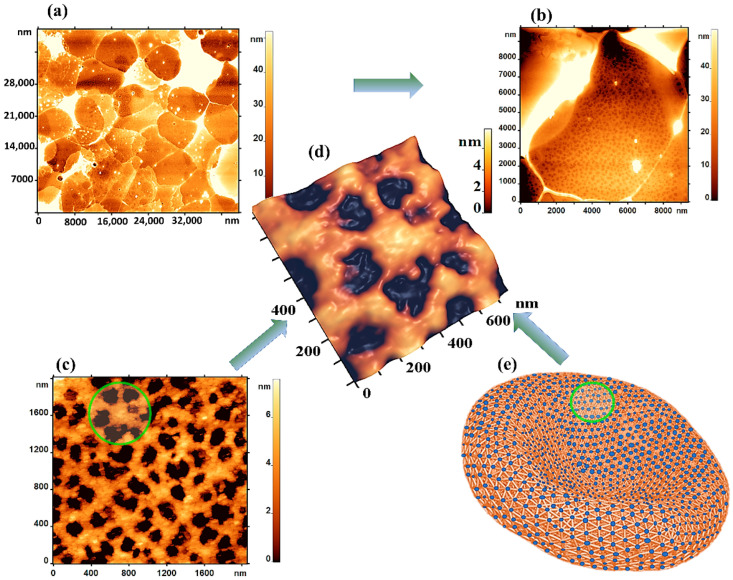
AFM images of the cytoskeleton: (**a**) Monolayer of ghost control cells (35 × 35 μm^2^); (**b**) Single RBC ghost (10 × 10 μm^2^); (**c**) Cytoskeleton section (1.8 × 1.6 μm^2^); (**d**) 3D AFM image of a section of the RBC cytoskeleton (0.8 × 0.7 μm^2^); (**e**) Model of the RBC cytoskeleton.

Cell morphology and elastic properties are determined by their cytoskeleton. Images of the RBC cytoskeleton network are shown in Figure 3. The thickness of the cytoskeleton network, represented as the experimentally obtained height of the pores above the substrate, was 5–8 nm.

The characteristic mesh size of the control sample was 150 ± 40 nm. Such a basic mesh was observed on 65 ± 20% of the outer surface of the ghosts, 5 ± 2% of the surface had a mesh with a characteristic size of more than 250 nm.

The cytoskeleton consists mainly of the spectrin protein, which is a fibrillar molecule 200–260 nm long and 2–3 nm thick. The subunits are arranged against each other, forming a helical structure. Dimers self-associate into tetramers and oligomers of a higher order. The tetramers bind to ankyrin, which in turn binds to the cytoplasmic domain, thus attaching the cytoskeleton to the plasma membrane. At its end, the tetramer binds the band 4.1 protein and the short actin filament, forming a network [24]. Figure 3e shows the model of the RBC cytoskeleton.

As a rule, each actin complex forms a spectrin network of 5–6 filaments (Figure 3d).

**Figure 4:**
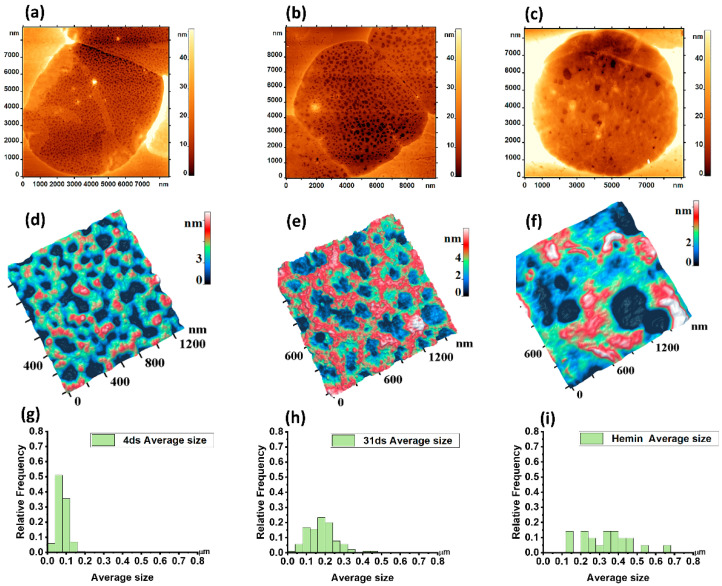
2D AFM image of a ghost cell: (**a**) Day 4 of storage; (**b**) Day 31 of storage; (**c**) After exposure to hemin. 3D AFM image of the cytoskeleton fragment: (**d**) Day 4 of storage; (**e**) Day 31 of storage; (**f**) After exposure to hemin. Histograms for the average size of ghost fragments are given: (**g**) Day 4 of storage; (**h**) Day 31 of storage; (**i**) After exposure to hemin.

The morphology of the cytoskeleton was analyzed using Image Analysis P9 software (NT-MDT Spectrum Instruments, Russia) utilizing the Advanced Watershed method. The average pore size of the control cells on day 4 of storage was 0.07 ± 0.02 μm. On day 31 of storage, the pore size was 0.18 ± 0.07 μm (Figure 4h). The number of pores per 2.5 × 2.5 μm^2^ area decreased from 300 ± 80 on day 4 of storage down to 85 ± 24 on day 31 of storage (*p* < 0.05).

Accordingly, the AFM image of the cytoskeleton fragment (Figure 4f) can be seen below the AFM image of the cell ghost exposed to hemin (Figure 4c). The discrete character of domains with topological nano defects seen as grains (Figure 2b) and their sizes correlated with the topological nanostructures of the cytoskeleton. The mean pore size ghost cells exposed to hemin was 0.32 ± 0.13 μm. The number of pores per 2.5 × 2.5 μm^2^ area was 30 ± 12 μm^−2^ (*p* < 0.05).

The distribution of mean pore size values in the controls were close to the Gaussian law. After hemin exposure and on day 31 of storage, the pRBC pores became larger in proportion to the deviation of the mean size from the normal distribution law. Thus, on day 31 of storage and after exposure to hemin, the mean and SD increased and non-normal data distribution with a skew to the right was observed. On day 31 day of storage, the pRBC distribution was slightly non-normal, whereas after hemin addition, the scattering of values of the main pore size increased greatly and the empirical histogram was not approximated by the Gaussian law.

## 4. Discussion

Since its invention in 1986 [43], the atomic force microscope has become one of the most important tools for visualizing biological objects at both the macro- and nano levels. Compared to light microscopy, which allows only investigation of RBC morphology and size, AFM helps to carry out the detailed study of the cell membrane structure. Identification of RBC membrane defects using the AFM in patients with systemic lupus erythematosus was reported [44], fibrinogen–erythrocyte interaction, as characterized by AFM-based force spectroscopy, alters in heart failure patients [30]. AFM imaging has shown that RBCs from healthy subjects and patients with multiple myeloma can be distinguished by morphological parameters and surface roughness [45].

In our study, altered morphology of RBCs was observed after their exposure to physical and chemical agents. Discocytes have been transformed into leptocytes, echinocytes, and stomatocytes (Figure 2). Topological defects emerged on the membrane surface (Figure 2), which could be the initiating mechanism of total cell damage. Using spectral-spatial Fourier transform of the nanosurface and RBC membranes, the native membrane parameters L and h were quantified. These parameters may be indicative of the structure of cell membranes. Changes in the L_1_ and h_1_ values provide information about the intensity of membrane flickering, while changes in the L_2_ and h_2_ parameters indicate changes in the cytoskeleton and protein aggregation. The mechanism of such changes is usually associated with alterations in the RBC cytoskeleton. During storage of the pRBCs, an image of the cytoskeleton with a size of 2.5 × 2.5 µm^2^ was obtained. The pore length of the cytoskeleton was shown to increase 2.17 times, and the number of pores decreased 5 times on day 31 vs day 4 of storage.

In our work, we also investigated the effect of oxidant (hemin) on the cytoskeleton structure. Using AFM, we observed the appearance of domains with topological nano defects manifested as grains in the RBC membranes after blood exposure to hemin. The correlation of the size of domains and typical dimensions of the cytoskeleton pore suggests that these topological nano defects are associated with altered cytoskeleton [46].

After exposure to hemin, the ghost cells with altered cytoskeleton were obtained to test this hypothesis. We observed distorted local domains in the cytoskeleton. Thus, we have shown in a direct biophysical experiment that hemin causes the destruction of the cytoskeleton network. We described a similar effect during the storage of erythrocyte suspension. The main abnormalities in the cytoskeletal reticulum consisted of disruption of the filaments, merging of small pores into larger ones, reduction in the number of pores, thickening or clusterization of filaments, and an increase in membrane stiffness [47,48]. There is a correlation between local topological nano defects in the RBC membranes and the change in the cytoskeleton nanostructure on the ghost cell surface. This suggests that domains with grain-like structures on the surface of membranes after exposure of RBCs to hemin are due to cytoskeleton damage (rupture and cluster formation).

The underlying mechanism of cytoskeletal changes may be associated with the following. Hemin causes the disruption of the spectrin—band 4.1 binding which breaks the connection of the spectrin filaments with band 3, and therefore with the lipid bilayer. Additionally, hemin oxidizes the spectrin molecules causing disruption of the connection between the dimers. Thus, the spectrin tetramers transform into dimers [49].

The mechanical properties of cells are important factors that determine cell function and mobility. In our experiments, Young’s modulus increased two and more times after exposure to pathogenic factors. Meanwhile, other authors [14,50] using atomic force spectroscopy showed that some diseases such as hypertension and diabetes mellitus are associated with reduced elasticity of erythrocytes which lose their capillary flow properties.

Understanding the differences between healthy and damaged RBCs is critical for diagnosing and treating various diseases. In critical states, severe blood loss or major operations donor blood can be required. An increased risk of post-transfusion complications associated with the use of long-stored pRBCs is one of the challenges of blood transfusion [51]. Using the AFM and AFS methods, it is possible to evaluate the quality of blood cells during the storage of erythrocyte suspension in a direct biophysical experiment [52].

Erythrocyte alterations can be used as diagnostic and prognostic tools in a wide range of conditions. The morphology of RBC is an important marker of human health. The ability of AFM to detect early subtle changes in the morphology, nanostructure, and cytoskeleton of erythrocytes opens up new perspectives for its use as a clinical screening tool.

## Figures and Tables

**Table 1:** Cell membrane parameters (L_1_, h_1_; L_2_, h_2_) of controls and cells exposed to hemin and zinc, and after 31 days of storage.

Exposure	L_1_, nm	h_1_, nm	L_2_, nm	h_2_, nm
Control	1133 ± 152 (1)	5.6 ± 1.5 (1)	90 ± 10 (1)	1.1 ± 0.5 (1)
Hemin	1109 ± 115 (0.97)	19.0 ± 4.0 (3.3)	152 ± 59 (1.7)	2.0 ± 0.6 (1.8)
Zinc	1092 ± 111 (0.96)	55.0 ± 16.0 (9.8)	202 ± 50 (2.2)	9.0 ± 1.5 (8.1)
Storage for 31 days	995 ± 194 (0.87)	9.1 ± 1.5 (1.6)	122 ± 32 (1.3)	3.3 ± 0.8 (3)

## Data Availability

The datasets used and analyzed during the current study are available from the corresponding authors on request.

## References

[1] Deng X., Xiong F., Li X., Xiang B., Li Z., Wu X., Guo C., Li X., Li Y., Li G. (2018). Application of atomic force microscopy in cancer research. J. Nanobiotechnol..

[2] Lal R., Ramachandran S., Arnsdorf M.F. (2010). Multidimensional Atomic Force Microscopy: A Versatile Novel Technology for Nanopharmacology Research. AAPS J..

[3] Yang F., Riedel R., del Pino P., Pelaz B., Said A.H., Soliman M., Pinnapireddy S.R., Feliu N., Parak W.J., Bakowsky U. (2017). Real-time, label-free monitoring of cell viability based on cell adhesion measurements with an atomic force microscope. J. Nanobiotechnol..

[4] Tian Y., Cai M., Xu H., Ding B., Hao X., Jiang J., Sun Y., Wang H. (2014). Atomic force microscopy of asymmetric membranes from turtle erythrocytes. Mol. Cells.

[5] Chang K.-C., Chiang Y.-W., Yang C.-H., Liou J.-W. (2012). Atomic force microscopy in biology and biomedicine. Tzu Chi Med. J..

[6] Sergunova V.A., Kozlova E.K., Myagkova E.A., Chernysh A.M. (2015). In vitro measurement of the elastic properties of the native red blood cell membrane. Gen. Reanimatol..

[7] Magonov S.N. (2015). Scanning Probe Based Apparatus and Methods for Low-Force Profiling of Sample Surfaces and Detection and Mapping of Local Mechanical and Electromagnetic Properties in Non-Resonant Oscillatory Mode. U.S. Patent.

[8] Alunda B.O., Lee Y.J. (2020). Review: Cantilever-Based Sensors for High Speed Atomic Force Microscopy. Sensors.

[9] Pi J., Cai J. (2019). Cell Topography and Its Quantitative Imaging by AFM. Atomic Force Microscopy. Methods in Molecular Biology.

[10] Abay A., Simionato G., Chachanidze R., Bogdanova A., Hertz L., Bianchi P., van den Akker E., von Lindern M., Leonetti M., Minetti G. (2019). Glutaraldehyde—A Subtle Tool in the Investigation of Healthy and Pathologic Red Blood Cells. Front. Physiol..

[11] Leyton-Puig D., Kedziora K.M., Isogai T., van den Broek B., Jalink K., Innocenti M. (2016). PFA fixation enables artifact-free super-resolution imaging of the actin cytoskeleton and associated proteins. Biol. Open.

[12] Pereira P.M., Albrecht D., Culley S., Jacobs C., Marsh M., Mercer J., Henriques R. (2019). Fix Your Membrane Receptor Imaging: Actin Cytoskeleton and CD4 Membrane Organization Disruption by Chemical Fixation. Front. Immunol..

[13] Kozlova E.K., Chernysh A.M., Moroz V.V., Kuzovlev A.N. (2013). Analysis of nanostructure of red blood cells membranes by space Fourier transform of AFM images. Micron.

[14] Lekka M., Fornal M., Pyka-Fościak G., Lebed K., Wizner B., Grodzicki T., Styczeń J. (2005). Erythrocyte stiffness probed using atomic force microscope. Biorheology.

[15] Ushiki T. (2001). Atomic force microscopy and its related techniques in biomedicine. Ital. J. Anat. Embryol..

[16] Lamzin I.M., Khayrullin R.M. (2014). The Quality Assessment of Stored Red Blood Cells Probed Using Atomic-Force Microscopy. Anat. Res. Int..

[17] Girasole M., Dinarelli S., Boumis G. (2012). Structure and function in native and pathological erythrocytes: A quantitative view from the nanoscale. Micron.

[18] Demchenkov E.L., Nagdalian A.A., Budkevich R.O., Oboturova N.P., Okolelova A.I. (2021). Usage of atomic force microscopy for detection of the damaging effect of CdCl2 on red blood cells membrane. Ecotoxicol. Environ. Saf..

[19] Ahyayauch H., García-Arribas A.B., Sot J., González-Ramírez E.J., Busto J.V., Monasterio B.G., Jiménez-Rojo N., Contreras F.X., Rendón-Ramírez A., Martin C. (2018). Pb(II) Induces Scramblase Activation and Ceramide-Domain Generation in Red Blood Cells. Sci. Rep..

[20] Marchesi V.T., Steers E. (1968). Selective Solubilization of a Protein Component of the Red Cell Membrane. Science.

[21] Elgsaeter A., Stokke B.T., Mikkelsen A., Branton D. (1986). The Molecular Basis of Erythrocyte Shape. Science.

[22] Byers T.J., Branton D. (1985). Visualization of the protein associations in the erythrocyte membrane skeleton. Proc. Natl. Acad. Sci. USA.

[23] Liu S.C., Derick L.H., Palek J. (1987). Visualization of the hexagonal lattice in the erythrocyte membrane skeleton. J. Cell Biol..

[24] Ursitti J.A., Pumplin D.W., Wade J.B., Bloch R.J. (1991). Ultrastructure of the human erythrocyte cytoskeleton and its attachment to the membrane. Cell Motil. Cytoskeleton.

[25] Girasole M., Dinarelli S., Boumis G. (2012). Structural, morphological and nanomechanical characterisation of intermediate states in the ageing of erythrocytes. J. Mol. Recognit..

[26] Pleskova S.N., Gorshkova E.N., Kriukov R.N. (2018). Dynamics of formation and morphological features of neutrophil extracellular traps formed under the influence of opsonized Staphylococcus aureus. J. Mol. Recognit..

[27] Liu F., Burgess J., Mizukami H., Ostafin A. (2003). Sample Preparation and Imaging of Erythrocyte Cytoskeleton with the Atomic Force Microscopy. Cell Biochem. Biophys..

[28] Encinar M., Casado S., Calzado-Martín A., Natale P., San Paulo Á., Calleja M., Vélez M., Monroy F., López-Montero I. (2017). Nanomechanical properties of composite protein networks of erythroid membranes at lipid surfaces. Colloids Surfaces B Biointerfaces.

[29] Buys A.V., Van Rooy M.-J., Soma P., Van Papendorp D., Lipinski B., Pretorius E. (2013). Changes in red blood cell membrane structure in type 2 diabetes: A scanning electron and atomic force microscopy study. Cardiovasc. Diabetol..

[30] Guedes A.F., Carvalho F.A., Malho I., Lousada N., Sargento L., Santos N.C. (2016). Atomic force microscopy as a tool to evaluate the risk of cardiovascular diseases in patients. Nat. Nanotechnol..

[31] Li M., Dang D., Xi N., Wang Y., Liu L. (2017). Nanoscale imaging and force probing of biomolecular systems using atomic force microscopy: From single molecules to living cells. Nanoscale.

[32] Lee G.-J., Park H.-K. (2015). Atomic force microscopy-based shape analysis of heart mitochondria. Methods Mol. Biol..

[33] Zhu Y. (2021). Atomic Force Microscopy Reveals the Role of Vascular Smooth Muscle Cell Elasticity in Hypertension. Atomic Force Microscopy—Basic Principles to Advanced Applications [Working Title].

[34] Kaul-Ghanekar R., Singh S., Mamgain H., Jalota-Badhwar A., Paknikar K.M., Chattopadhyay S. (2009). Tumor suppressor protein SMAR1 modulates the roughness of cell surface: Combined AFM and SEM study. BMC Cancer.

[35] Chernysh A.M., Kozlova E.K., Moroz V.V., Sergunova V.A., Gudkova O.E., Kozlov A.P., Manchenko E.A. (2018). Nonlinear Local Deformations of Red Blood Cell Membranes: Effects of Toxins and Pharmaceuticals (Part 2). Gen. Reanimatol..

[36] Cynober T., Mohandas N., Tchernia G. (1996). Red cell abnormalities in hereditary spherocytosis: Relevance to diagnosis and understanding of the variable expression of clinical severity. J. Lab. Clin. Med..

[37] Krol A.Y., Grinfeldt M.G., Levin S.V., Smilgavichus A.D. (1990). Local mechanical oscillations of the cell surface within the range 0.2–30 Hz. Eur. Biophys. J..

[38] Jones A.R., Patel R.P., Marques M.B., Donnelly J.P., Griffin R.L., Pittet J.-F., Kerby J.D., Stephens S.W., DeSantis S.M., Hess J.R. (2019). Older Blood Is Associated With Increased Mortality and Adverse Events in Massively Transfused Trauma Patients: Secondary Analysis of the PROPPR Trial. Ann. Emerg. Med..

[39] Kozlova E., Chernysh A., Manchenko E., Sergunova V., Moroz V. (2018). Nonlinear biomechanical characteristics of deep deformation of native RBC membranes in normal state and under modifier action. Scanning.

[40] Sherstyukova E.A., Inozemtsev V.A., Kozlov A.P., Gudkova O.E., Sergunova V.A. (2021). Atomic force microscopy in the assessment of erythrocyte membrane mechanical properties with exposure to various physicochemical agents. Alm. Clin. Med..

[41] Turrini F., Mannu F., Arese P., Yuan J., Low P.S. (1993). Characterization of the autologous antibodies that opsonize erythrocytes with clustered integral membrane proteins. Blood.

[42] Harmaza Y.M., Tamashevski A.V., Slobozhanina E.I. (2020). Molecular nature of cytotoxicity of zinc oxide nanostructures. Dokl. Natl. Acad. Sci. Belarus.

[43] Binnig G., Quate C.F., Gerber C. (1986). Atomic Force Microscope. Phys. Rev. Lett..

[44] Kamruzzahan A.S.M., Kienberger F., Stroh C.M., Berg J., Huss R., Ebner A., Zhu R., Rankl C., Gruber H.J., Hinterdorfer P. (2004). Imaging morphological details and pathological differences of red blood cells using tapping-mode AFM. Biol. Chem..

[45] Zhang Y., Zhang W., Wang S., Wang C., Xie J., Chen X., Xu Y., Mao P. (2012). Detection of Erythrocytes in Patients With Multiple Myeloma Using Atomic Force Microscopy. Scanning.

[46] Kozlova E., Chernysh A., Moroz V., Gudkova O., Sergunova V., Kuzovlev A. (2014). Transformation of membrane nanosurface of red blood cells under hemin action. Sci. Rep..

[47] Kozlova E., Chernysh A., Moroz V., Kozlov A., Sergunova V., Sherstyukova E., Gudkova O. (2021). Two-step process of cytoskeletal structural damage during long-term storage of packed red blood cells. Blood Transfus..

[48] Sherstyukova E., Chernysh A., Moroz V., Kozlova E., Sergunova V., Gudkova O. (2021). The relationship of membrane stiffness, cytoskeleton structure and storage time of pRBCs. Vox Sang..

[49] Mohandas N., Gallagher P.G. (2008). Red cell membrane: Past, present, and future. Blood.

[50] Shin S., Ku Y., Babu N., Singh M. (2007). Erythrocyte deformability and its variation in diabetes mellitus. Indian J. Exp. Biol..

[51] Garraud O. (2017). Effect of “old” versus “fresh” transfused red blood cells on patients’ outcome: Probably more complex than appears. J. Thorac. Dis..

[52] Kozlova E., Chernysh A., Moroz V., Sergunova V., Gudkova O., Manchenko E. (2017). Morphology, membrane nanostructure and stiffness for quality assessment of packed red blood cells. Sci. Rep..

